# American Medical Association

**Published:** 1859-04

**Authors:** 


### EDITORIAL.

### AMERICAN MEDICAL ASSOCIATION.

The next meeting of this society is to take place at Louisville on the first Tuesday of May next. On the day preceding, there is to be a convention of medical teachers, to take into consideration the question of certain alterations of the present plan of teaching, which have been proposed, and which it is fashionable to call medical reform. Almost every journal among our exchanges has had a word to say on the subject of the action of this convention, and it seems to be expected in some quarters, that it will be able to propose some kind of alteration, which is to be of signal benefit to the profession. For ourselves, we regard this expectation as altogether unreasonable.

In the first place, neither the convention, nor the association, has any power over the action of the colleges, the profession, the public, or the students.

The colleges will go on as heretofore, granting diplomas so long as it is for their interest so to do.

The profession will, as before, remain, for the most part, indifferent spectators of the action of the colleges.

The public will continue to employ the doctors graduated by the colleges, so long as they are the best they can get. When the people will no longer employ the doctors created by the colleges, the conferring degrees will be likely to be given up.

Students will continue to resort to the best or the most popular schools, or, if of limited means, to those within their reach.

How, then, is medical instruction to be improved? This must be done by substituting the idea of improvement for that of "reform." This is to be effected by improving the quality of the lectures in the medical schools. Excellent as are many of these courses, there is still room for improvement, particularly in the means of illustration. What is necessary for teaching is also indispensable for investigating. The founding of the Mott and Mutter collections is among the best evidences of progress, and the most cheering signs of the times. Medical libraries adapted to the purpose of the thorough research are among the means of improvement whose want is most severely felt.

Teaching outside of colleges, in offices, hospitals, and by associations, is to be encouraged. What would the medical teaching of Paris be if reduced to the official courses? It is the private courses which give to the teaching of that city its greatest advantages. In this respect, the improvement which has taken place in this country within the last twenty years, is remarkable. Twenty years ago, it was difficult to obtain a thorough knowledge of auscultation and percussion, still less of other *specialities*, in the United States. Now, there is no large city in which they cannot be acquired with a good degree of accuracy.

Still, there is much to be desired in this respect, and it is in the power of the colleges to effect something in the way of improvement, by instituting preparatory and complementary courses of teaching.

Already have movements in the right direction been made, and it is from their encouragement that improvements in teaching can be expected.

So long as the cant phrase "elevating the profession," is the motto, little is to be expected in the way of true scientific advancement. The physician who, by diligent study, acquires skill and knowledge beyond his neighbors, not only receives the reward of his diligence, but stimulates many others to imitate his example. He who writes an essay of value to the profession, not only improves himself and instructs others, but sets an example, the influence of which is felt far and wide. The same is more emphatically true of valuable books. General improvement is composed of an aggregate of individual instances of it; and these latter must precede the former.

If any man mourns once the low state of medical knowledge, let him by his own industry, set an example of what a physician should be in practice, teaching and writing. That is the best thing he can do.

We do not despair of seeing something accomplished by the national asssociation much more useful than anything it has yet done. It is discouraging to see incompetent, noisy individuals thrusting themselves into places of prominence in its meetings. It is melancholy to read some of the reports on "medical literature," and medical education, which these same persons, as chairmen of committees, have inflicted upon us. But these are rather the exceptions than the rule.

We indulge the hope of yet seeing the association so organized, that the presenting and discussion of scientific papers and subjects, in sections, shall constitute the business of its meetings. Of how great advantage to the surgeons and surgery of America would a meeting of those cultivating that branch of the science be, if assembled for discussion, we need not say. To us, this appears to be at the present moment, the only suggestion in the way of reform which we feel at liberty to make.

We omitted to mention, that Taylor's work on Poisons can be procured of W. B. Keen, 148 Lake street, who keeps a large assortment of medical works.

We are making arrangements to issue the *Journal*, on the first of every month. On the first of June, we expect to furnish our patrons at the proper time.

Those who have not paid in full are requested to forward the amount in arrears, as this will save the expense of collecting. We feel satisfied that they will gladly comply with this request.

☞ In future the space devoted to advertisements will be an addition to this *Journal*, and not be taken from the body of the work. The advertising department is now placed in the hands of the printer, Mr. J. Barnet, 189 Lake street.

